# Quantitative analysis of retinal microvascular changes in macular telangiectasia type 2 using optical coherence tomography angiography

**DOI:** 10.1371/journal.pone.0232255

**Published:** 2020-04-29

**Authors:** Young Gun Park, Young-Hoon Park

**Affiliations:** 1 Department of Ophthalmology and Visual Science, Seoul St. Mary’s Hospital, College of Medicine, The Catholic University of Korea, Seoul, Korea; 2 Catholic Institute for Visual Science, Collage of Medicine, The Catholic University of Korea, Seoul, Korea; Massachusetts Eye & Ear Infirmary, Harvard Medical School, UNITED STATES

## Abstract

**Purpose:**

To evaluate retinal vascular changes on optical coherence tomography angiography (OCTA) in patients with macular telangiectasia type 2 (MacTel 2) and to assess their correlation with visual acuity.

**Methods:**

Twenty-six patients (52 eyes) with MacTel 2 and 20 age-matched controls (40 eyes) were included. Fundus examinations, including fundus autofluorescence, swept-source optical coherence tomography, and OCTA, were performed. Differences in the vascular density in the fovea and parafovea, the area of the foveal avascular zone, and the diameter of the ellipsoid zone defect of the two groups were analyzed.

**Results:**

The foveal vascular density of the superficial capillary plexus was significantly lower in the MacTel 2 group than in the control group (p = 0.027). The vascular density in the entire deep capillary plexus was also significantly less in the MacTel 2 group than in the control group (all p < 0.05). The mean diameter of ellipsoid-zone disruption on OCT in the MacTel 2 group was 634.6 ± 104.3 𝜇m. The foveal avascular zone areas of the superficial and deep capillary plexuses were significantly enlarged in the MacTel 2 group compared to those in the control group (0.45 ± 0.12 mm^2^ vs. 0.27 ± 0.08 mm^2^, p < 0.001; 0.56 ± 0.15 mm^2^ vs. 0.40 ± 0.14 mm^2^, p = 0.001). In addition, the enlarged foveal avascular zone of the superficial and deep plexus was negatively correlated with best corrected visual acuity (logMAR) in MacTel 2 patients (p = 0.013, r = -0.642 and p = 0.042, and r = -0.511, respectively).

**Conclusions:**

Retinal vascular density changes occur in the superficial fovea and in the entire deep capillary plexus of patients with MacTel 2. The enlarged foveal avascular zone areas of the superficial and deep plexuses were prominent in the MacTel 2 group, and this enlargement correlates with worsened visual acuity.

## Introduction

Macular telangiectasia type 2 (MacTel 2) is a neurodegenerative disease related to Müller cell dysfunction and vascular alterations in the macular capillary network [1, 2]. Patients with this condition have loss of macular transparency surrounding the fovea, crystalline deposits in the inner retina, right-angled vessels, and intraretinal cavities [3, 4]. These typical changes occur along with thinning of the central retina and focal loss of the ellipsoid zone (EZ). Later stages of the disease involve migration of the hyperplastic retinal pigment epithelium, loss of macular pigment, and development of a subretinal neovascular membrane [5, 6].

Fundus fluorescein angiography (FFA) has been widely used as a standard diagnosis method in patients with MacTel 2. Typical FFA findings show ectatic vascular changes with hyper-fluorescence in the parafoveal area [3]. In addition, optical coherence tomography (OCT) can also depict the morphological changes in MacTel 2 [7]. OCT has been used to show neurodegenerative changes, including thinning of the central retina, cavitations in the inner or outer retina, and focal loss in the EZ from the temporal side to the foveal center, then progressively through these breaks in the retinal pigment epithelium, extending to the nasal macula. It finally leads to central EZ loss and foveal atrophy.[6, 8] The presence of EZ loss on OCT is an important factor for the prognosis of patients with MacTel 2 [9].

Optical coherence tomography angiography (OCTA) is a new method used to visualize the choroidal and retinal vasculature and blood-flow map that does not require dye injection. OCTA can selectively visualize specific layers—the superficial vascular plexus, the deep vascular plexus, and the outer capillary plexus—which is in contrast with fluorescein angiography. This information can help clarify the progressive changes and visual prognosis in patients with MacTel 2 [10, 11]. We compared the superficial and deep retinal vascular density and the foveal avascular zone (FAZ) using OCTA and compared their correlations with visual acuity in the MacTel 2 and control groups.

## Materials and methods

This study was a retrospective review of consecutive cases. Patients attending the Department of Ophthalmology of Seoul St. Mary’s Hospital in Seoul, Korea, between January 2017 and January 2019 with a confirmed diagnosis of MacTel type 2 were included. All procedures were conducted in accordance with the Declaration of Helsinki (1964) and its later amendments. The study was approved by the ethics committee of Seoul St. Mary’s Hospital, The Catholic University of Korea. The need to obtain informed patient consent was waived due to the retrospective design of the study.

All patients and controls underwent standardized dilated fundus examinations, including measurements of best-corrected visual acuity (BCVA), swept-source OCT (SS-OCT), and OCTA imaging (DRI OCT Triton, Topcon, Japan). We performed fundus photography, fundus autofluorescence (FAF), and FFA for patients with MacTel type 2 to confirm their diagnosis. The overall disease severity of included participants corresponded to stages 1–5, as described by Gass and Blodi [12]. The exclusion criteria were preexisting macular disease (e.g. epiretinal membrane or any macular dystrophy) or severe media opacity (e.g. lens opacity due to cataract or thick asteroid hyalosis). The control group included healthy patients attending medical checkups; they had no posterior segment abnormalities or systemic co-morbidities. Their health promotion programs involved both necessary and optional examinations, including OCT and OCTA.

### Swept-source optical coherence tomography

SS-OCT (DRI OCT Triton, Topcon, Tokyo, Japan) was performed using an axial scan rate of 100,000 Hz, with a laser wavelength of 1050 nm, yielding an 8-μm axial resolution and 20-μm transverse resolution. All subjects underwent 12 mm × 9 mm radial and 5-line scans centered at the fovea. Patients with low-quality images were excluded. Using foveal SS-OCT images, the presence or absence (and, if present, the length) of EZ loss was determined by two investigators (YGP, YHP), who were blinded to each other.

### Optical coherence tomography angiography

OCTA was performed with a DRI OCT Triton (Topcon). This instrument has an A-scan rate of 70,000 scans/s with an 840-nm wavelength light source and a 45-nm bandwidth. Patients with low-quality images were excluded. OCTA images were evaluated using automatic segmentation; we analyzed the vascular density of the superficial and deep retinal vascular zone using computer software. The extent of the FAZ was also manually measured on the OCTA images of each participant by the two investigators (YGP, YHP) who were blinded to each other.

### Statistical analysis

Paired *t*-tests were used to compare the groups, and Pearson’s correlation coefficients were used for correlation analysis. P < 0.05 was considered statistically significant. All analysis were produced using commercial software (version 22.0; SPSS Statistics, Inc. Chicago, IL, USA).

## Results

**Fig 1:**
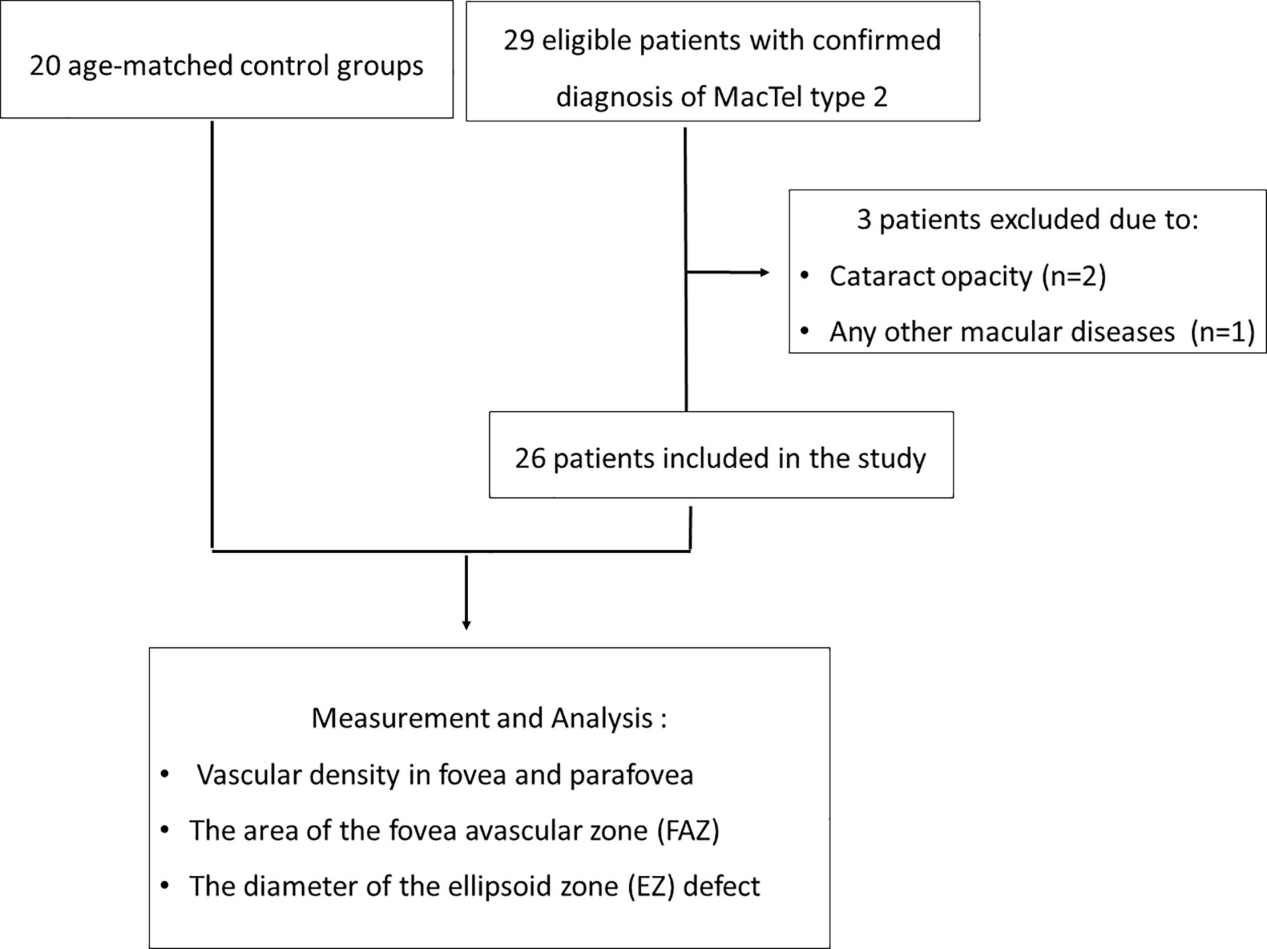
Study flow chart.

**Fig 2:**
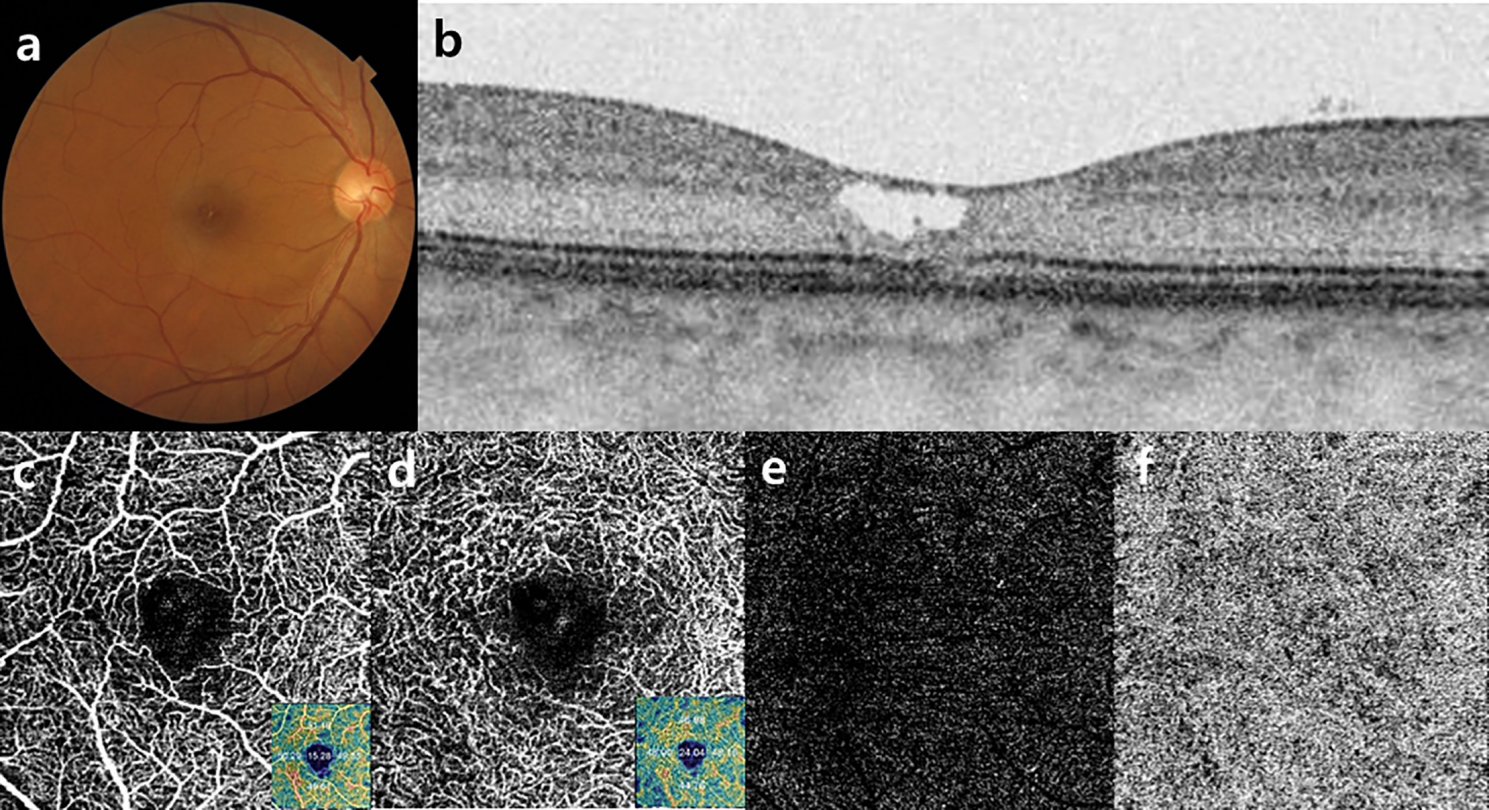
Representative images of an eye with MacTel 2. (a, b) Fundus photograph showing loss of retinal transparency from the temporal side to the fovea and focal loss of the ellipsoid zone on spectral domain optical coherence tomography images. (c–f) Vascular density map of an eye with MacTel 2 with optical coherence tomography angiography (c, superficial capillary plexus; d, deep capillary plexus; e; outer retina; f, choriocapillaris).

Twenty-six patients (52 eyes) with MacTel 2 and 20 age-matched control subjects (40 eyes) were finally included (Fig 1). The mean age of patients with MacTel 2 was 66.6 ± 5.9 years; that of the controls was 64.9 ± 9.4 years (p = 0.531). The distribution of cases according to the stage of disease was as follows: stage 1, 3.8% (2/52); stage 2, 36.5% (19/52); stage 3, 40.4% (21/52); stage 4, 15.4% (8/52); and stage 5, 3.8% (2/52). Representative images of the retinal microvasculature in a patient with MacTel 2 are shown in Fig 2.

The foveal vascular density of the superficial plexus was significantly lower in the MacTel 2 group than in the control group (13.27% vs. 15.29%, p = 0.027). The parafoveal vascular density in the superficial plexus did not differ significantly between the two groups (superior/temporal/inferior/nasal; all p > 0.05). The foveal vascular density in the deep capillary plexus was also significantly decreased in the eyes with MacTel 2 (22.58 ± 6.98%) than in the controls (32.25 ± 8.95%; p = 0.001). All vascular density parameters for the deep capillary plexus were significantly lower in the MacTel 2 group than in the control group (superior/temporal/inferior/nasal: p = 0.001, p = 0.006, p = 0.027, p = 0.035, and p = 0.017, respectively; Table 1). Rarefaction of the whole deep capillary network was identified in patients with MacTel 2. Only foveal vascular density in the superficial capillary plexus was lower in the MacTel 2 group than in the control group. This suggests a relationship between disease progression and the direction of vascular changes.

**Table 1:** Foveal and parafoveal vessel density of the superficial and deep plexuses evaluated by optical coherence tomography angiography in the MacTel 2 group and control group.

	MacTel 2 Group	Control Group	P value
Superficial Plexus (%)			
Fovea	13.27 ± 4.47	15.29 ± 3.53	0.027*
Superior	48.53 ± 4.79	48.60 ± 7.90	0.590
Temporal	45.50 ± 3.25	47.98 ± 6.13	0.057
Inferior	46.94 ± 5.47	49.44 ± 5.35	0.614
Nasal	42.82 ± 3.67	46.06 ± 4.72	0.050
Deep Plexus (%)			
Fovea	22.58 ± 6.98	32.25 ± 8.95	0.001*
Superior	43.75 ± 5.50	48.75 ± 6.29	0.006*
Temporal	43.84 ± 5.30	49.79 ± 6.57	0.027*
Inferior	41.13 ± 6.97	45.42 ± 6.89	0.035*
Nasal	43.67 ± 5.45	47.71 ± 6.34	0.017*

The mean diameter of EZ disruption in patients with MacTel 2 on OCTA was 634.6 ± 104.3 μm; this parameter was not correlated with visual acuity. The association of BCVA loss was affected by central involvement.[6, 8] However, the FAZ areas of the superficial and deep plexuses were significantly enlarged in the MacTel 2 group when compared with those in the control group (0.45 ± 0.12 mm^2^ vs. 0.27 ± 0.08 mm^2^, p < 0.001; 0.56 ± 0.15 mm^2^ vs. 0.40 ± 0.14 mm^2^, p = 0.001, respectively; Table 2). There was a statistically significant negative correlation between the FAZ areas in the superficial and deep capillary plexuses and BCVA (logMAR) in the MacTel 2 group (r = -0.642, p = 0.013; and r = -0.511, p = 0.042, respectively; Table 3).

**Table 2:** Foveal avascular zone (FAZ) area (mm^2^) of the superficial and deep plexuses determined by optical coherence tomography angiography in the MacTel 2 group and control group.

	MacTel 2 Group	Control Group	P value
Superficial FAZ	0.45 ± 0.12	0.27 ± 0.08	<0.001 *
Deep FAZ	0.56 ± 0.15	0.40 ± 0.14	0.001 *

**Table 3:** Correlation between optical coherence tomography angiography and visual acuity in patients with MacTel type 2.

	Correlation constant	P value
Superficial plexus		
Fovea	0.035	0.806
Superior	-0.032	0.822
Temporal	-0.174	0.218
Inferior	-0.244	0.081
Nasal	0.081	0.610
Foveal avascular zone area	-0.642*	0.013*
Deep plexus		
Fovea	0.104	0.464
Superior	0.117	0.407
Temporal	-0.057	0.088
Inferior	-0.080	0.573
Nasal	0.165	0.242
Foveal avascular zone area	-0.511*	0.042*

## Discussion

OCTA can selectively visualize specific retinal layers, which can help to clarify the pathophysiology and progressive retinal changes in patients with MacTel 2 [10, 11]. We therefore compared the OCTA-based superficial and deep retinal vascular density and FAZ in patients with MacTel 2 and in control subjects, and assessed the correlation of these changes with visual prognosis. We demonstrated that significant retinal vascular density changes occur in the superficial fovea and entire deep capillary plexus in patients with MacTel 2. These patients also showed enlarged FAZ areas in the superficial and deep plexuses, and the enlargement correlated with worse visual prognosis.

MacTel 2 is a bilateral, neurodegenerative, and neurovascular disease that causes some alteration in the juxtafoveolar capillary network [1, 13]. Although FFA is the gold standard diagnostic method, it may be insufficient to show changes in specific retinal layers and in the vasculature and to describe pathologic abnormalities. Peto et al. showed that OCT could reveal a relationship between loss of visual acuity and disruption of the EZ [8]. They demonstrated that EZ loss may be the best currently available parameter for monitoring MacTel progression; when the EZ loss progressed to the foveal center, visual acuity was clinically affected. Heeren et al. also reported that the extent of EZ loss correlated significantly with a change in scotomas, but not with loss of BCVA [14]. This is in accordance with our finding that the diameter of EZ loss was not directly correleted with BCVA, perhaps because visual loss can result from a variety of factors other than foveal involvement.

OCTA imaging facilitates detection of abnormal vascular structures in the parafoveal region, which assists in the diagnosis of Mactel 2 and monitoring its progression [10, 15–17]. Toto et al. reported that foveal vascular density, both in the superficial and deep plexuses, was decreased in 15 eyes with MacTel 2 and found a decrease in parafoveal vessel density only in the superficial plexus [16]. In contrast, Spaide et al. showed that there was loss of capillaries in both the superficial and deep plexuses in the macular region, which was more marked in the deep plexus [18]. They found that patients with advanced stages had more prominent decreases in vascular density in the deep plexus and mildly increased loss of the perifoveal capillaries of the superficial plexus. Patients with MacTel 2 may not have typically detectable manifestations until the disease reaches the advanced stage. In the present study, eyes with MacTel 2 appeared to have broad areas of abnormalities in the outer retinal vascular plexus around the macula, based on OCTA.

**Fig 3:**
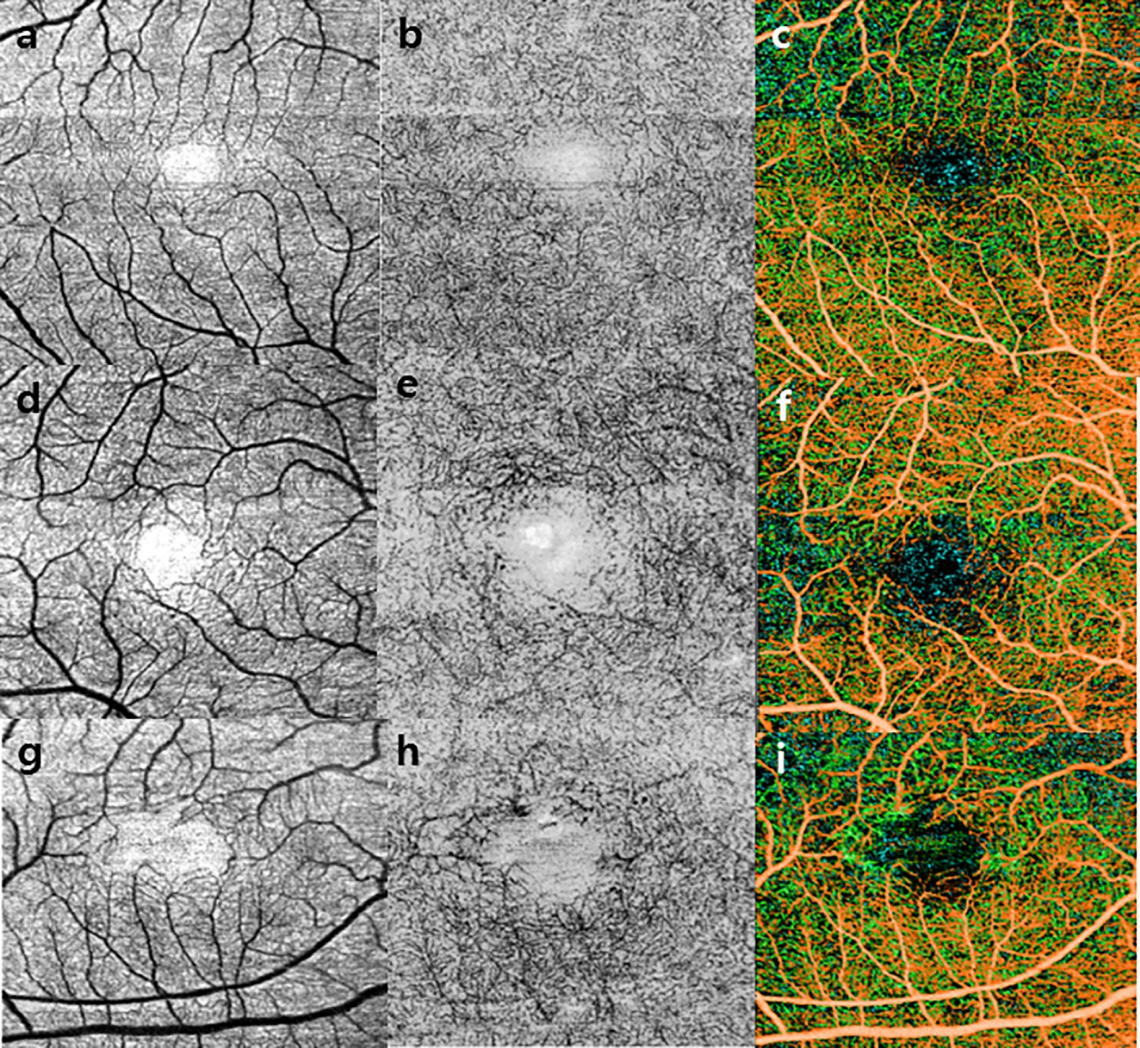
Changes in the foveal avascular zone (FAZ) between MacTel 2 and control eyes as seen on optical coherence tomography angiography. (a) Superficial capillary plexus; (b) deep capillary plexus; and (c) color stack images of normal eyes; (d–f) the same images of an eye with early stage MacTel 2 and (g–i) an eye with advanced MacTel 2.

We also found that the vascular density significantly decreased in patients with Mactel 2. In particular, the entire retinal vascular density of the deep plexus was significantly lower in the Mactel 2 group than in the control group (fovea/superior/temporal/inferior/nasal; p = 0.001, p = 0.001, p = 0.006, p = 0.027, p = 0.035, and p = 0.017, respectively; Fig 3). However, only the foveal vascular density of the superficial plexus differed significantly between the two groups (13.27% vs. 15.29%, p = 0.027). Thus, changes in vascular density reflected loss of the capillary vascular network as well as telangiectatic vessels, particularly in the deep capillary plexus. We detected a decrease in the entire vascular density only in the deep plexus, and changes in the superfical capillaries were detected only in the fovea, not in the parafovea. The present series demonstrated that changes occurred distinctly in the whole deep capillary plexus, rather than in the superficial capillary plexus.

The retinal vascular and other abnormalities seen in MacTel 2 patients may be the result of abnormal function prior to the death of Müller cells [19–22]. Müller cells play important roles in a protective function for both vascular cells and retinal neurons [23–27]. They also regulate retinal blood flow and angiogenesis. Therefore, Müller-cell degeneration may lead to progressive changes in retinal blood vessels, including the loss of capillary density. This finding differs from the previous proposal by Gass et al. that the telangiectatic vessels altered the capillary walls, which then caused deterioriation of metabolic exchange [12]. Recently, Müller-cell abnormalities in patients with MacTel2 have been suggested to be the initial pathology, which subsequently progresses to retinal atrophy with vascular changes [13, 28]. In the present study, eyes with MacTel 2 appeared to have abnormalities in the outer retinal vascular plexus around macula, as determined by OCTA.

In our study, the FAZ areas of the superficial and deep plexuses were found to be significantly larger in the MacTel 2 group than in the control group (p < 0.001 and p = 0.01, respectively). A statistically significant negative correlation was found between the FAZ areas of the superficial and deep plexuses and visual acuity in MacTel 2 patients. We found a negative correlation between BCVA and the area of the FAZ, not any of the other parameters. One possible explanation for this finding is that foveal vessel rarefaction and large foveal cavitations with retinal atrophy are related to enlargement of the FAZ area. The changes of the FAZ area may be associated with outer retinal disorganization and contributed to the progressive deterioration of BCVA. Interestingly, we found a negative correlation between the FAZ areas in both the superficial and deep vascular networks and visual acuity; these correlations were independent of the diameter of EZ loss (r = -0.642, p = 0.013; and r = -0.511, p = 0.042, respectively). The correlation with the superficial FAZ area was slightly stronger than that of the deep FAZ area. However, we did not find much difference between the two layers, and further cases are necessary to prove this.

In accordance with this, the loss of the capillary vascular plexus would lead to the FAZ enlargement seen on OCTA; this may play an important role in the photoreceptor and outer retinal changes seen in patients with MacTel 2. Structural degradation, which suggests photoreceptor loss, heralds secondary clinical changes and is related to functional loss. This finding suggests that the earliest changes are apparent on OCTA, before manifestations that correlate with vision loss. These are specific quantitative parameters for analysis at follow-up.

The limitations of our study included its retrospective design and the small sample size. No significant comparison was able to be performed between stages since there were not enough patients with different stages. And this study was conducted in a cross-sectional manner and future collection of longitudinal data is needed in order to verify our results. However, to our knowledge, no previous study has demonstrated the correlation between retinal vascular density, the FAZ, and visual acuity in MacTel 2 patients.

In conclusion, we observed changes in the retinal vascular density of the deep capillary plexus and the foveal vasccular density of superficial plexus and an enlarged FAZ in patients with Mactel 2. We found a significant negative correlation between the FAZ in the superficial plexus and visual acuity. OCTA has the typical advantages of OCT imaging, including the ability to detect abnormal microvascular structures and to monitor the progression of these abnormalities. Our study demonstrated that the earliest changes were observed in the deep capillary plexus, parafovea, and temporal vascular density and FAZ of the deep capillary plexus. Further studies are required to confirm the predictive value of these findings.

## Supporting information

S1 Fig (TIF)
