# Supplementary figures and images for: Quantitative analysis of retinal microvascular changes in macular telangiectasia type 2 using optical coherence tomography angiography
Source: PLoS One. 2020 Apr 29;15(4):e0232255. doi: 10.1371/journal.pone.0232255 (PMC7190136; doi:10.1371/journal.pone.0232255)

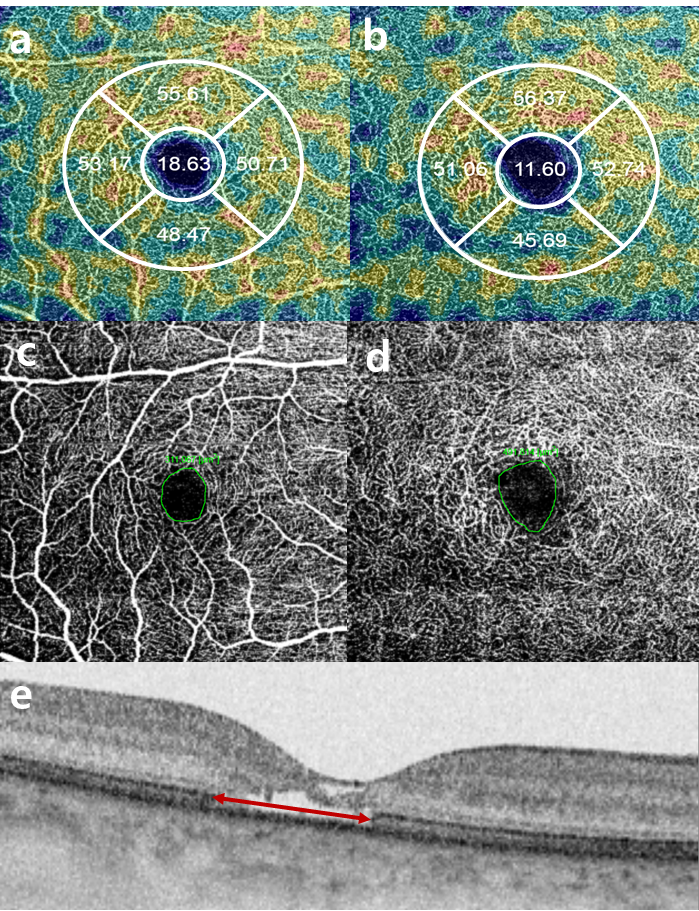

Supplement: S1 Fig — (TIF) [file pone.0232255.s001.tif]
